# Eldecalcitol prevents muscle loss and osteoporosis in disuse muscle atrophy via NF-κB signaling in mice

**DOI:** 10.1186/s13395-023-00332-0

**Published:** 2023-12-19

**Authors:** Haichao Zhang, Yanping Du, Wenjing Tang, Minmin Chen, Weijia Yu, Zheng Ke, Shuangshuang Dong, Qun Cheng

**Affiliations:** 1 Department of Osteoporosis and Bone Disease, Huadong Hospital Affiliated to Fudan University, Research Section of Geriatric Metabolic Bone Disease, Shanghai Geriatric Institute, Shanghai, 200040 People’s Republic of China; 2 Medical Division, Chugai Pharma China Co., Ltd., Shanghai, 200021 People’s Republic of China

**Keywords:** Eldecalcitol, Tail suspension, Grip strength, Vitamin D receptor, NF-κB signaling

## Abstract

We investigated the effect of eldecalcitol on disuse muscle atrophy. C57BL/6J male mice aged 6 weeks were randomly assigned to control, tail suspension (TS), and TS-eldecalcitol–treated groups and were injected intraperitoneally twice a week with either vehicle (control and TS) or eldecalcitol at 3.5 or 5 ng for 3 weeks. Grip strength and muscle weights of the gastrocnemius (GAS), tibialis anterior (TA), and soleus (SOL) were determined. Oxidative stress was evaluated by malondialdehyde, superoxide dismutase, glutathione peroxidase, and catalase. Bone microarchitecture was analyzed using microcomputed tomography. The effect of eldecalcitol on C2C12 myoblasts was analyzed by measuring myofibrillar protein MHC and the atrophy markers Atrogin-1 and MuRF-1 using immunofluorescence. The influence of eldecalcitol on NF-κB signaling pathway and vitamin D receptor (VDR) was assessed through immunofluorescence, (co)-immunoprecipitation, and VDR knockdown studies. Eldecalcitol increased grip strength (*P* < 0.01) and restored muscle loss in GAS, TA, and SOL (*P* < 0.05 to *P* < 0.001) induced by TS. An improvement was noted in bone mineral density and bone architecture in the eldecalcitol group. The impaired oxidative defense system was restored by eldecalcitol (*P* < 0.05 to *P* < 0.01 vs. TS). Eldecalcitol (10 nM) significantly inhibited the expression of MuRF-1 (*P* < 0.001) and Atrogin-1 (*P* < 0.01), increased the diameter of myotubes (*P* < 0.05), inhibited the expression of P65 and P52 components of NF-κB and P65 nuclear location, thereby inhibiting NF-κB signaling. Eldecalcitol promoted VDR binding to P65 and P52. VDR signaling is required for eldecalcitol-mediated anti-atrophy effects. In conclusion, eldecalcitol exerted its beneficial effects on disuse-induced muscle atrophy via NF-κB inhibition.

## Introduction

Muscle atrophy is a progressive and systemic disease caused by multiple factors. When muscles are inactive for a prolonged period, muscle proteins are lost causing muscle fibers to deteriorate [30]. Denervation and various conditions including endocrine diseases (such as diabetes and hypogonadism), osteoporosis, and cancer can also result in muscle atrophy [5, 35]. In addition, loss of muscle mass and function can lead to an increase in morbidity and mortality along with a decrease in quality of life [10]. Muscle loss due to prolonged inactivity frequently occurs during continued bed rest, sedentary lifestyle, limb immobilization, and spaceflight [6]. Patients with either chronic disease or cachexia (cancer-induced) or aging are also prone to the development of skeletal muscle atrophy [5, 7, 29, 37, 38]. Furthermore, women with disuse atrophy have been shown to experience ICU frailty at higher rates compared with men [19]. Aging and physical inactivity adversely affect lean mass and muscle force production [2]. Disuse muscle atrophy has received much interest owing to the growing aging population. Exercise is the most effective way to fight against muscle atrophy, as there is no effective pharmacological treatment [5].

The transcription factor, nuclear factor kappa-B (NF-κB) is an upstream regulator of inflammatory responses and other key biological processes [42]. Moreover, NF-κB and its family members play an important role in muscle atrophy attributed to both disuse and cachexia [13, 15]. Several NF-κB family members are upregulated during disuse atrophy, which stimulates downstream target genes involved in inflammation and protein turnover [3]. In normal conditions, the inhibitor of κB proteins inhibits NF-κB DNA-binding activity and nuclear location. After hind limb unloading, NF-κB levels increase considerably along with atrophy-linked E3 ubiquitin ligase, muscle-specific RING finger-1 (MuRF-1) and Atrogin-1, which then induce proteasomal degradation [40].

Vitamin D is regarded as an essential factor for preventing and treating osteoporosis. It exerts its main cellular effects by activating the vitamin D receptor (VDR). Eldecalcitol, a 2β-hydroxypropoxylated analog of active vitamin D, has a higher affinity for vitamin D–binding protein than 1α,25-hydroxyvitamin D, which may explain its prolonged half-life in serum [26]. A previous study reported that eldecalcitol increased bone mineral density (BMD) in the femoral metaphysis by suppressing bone resorption [11]. Furthermore, many clinical studies have shown that eldecalcitol can also improve muscle strength in postmenopausal women [32, 33]. However, there are no studies on the direct effects of eldecalcitol on disuse muscle atrophy. There is an unmet therapeutic need in the treatment of muscle atrophy. Hence, to develop an optimal therapeutic agent, we need to understand the effects of eldecalcitol on the signaling pathway that occurs during the process of muscle atrophy.

Tail suspension (TS) is a classical foundational model to study disuse muscle atrophy. Due to the difficulty involved in mimicking prolonged muscle disuse (bed rest, spaceflight, etc) in humans under controlled conditions, the hind limb suspension/hind limb unloading model was developed in rodents and is an appropriate model to study disuse-induced skeletal muscle atrophy [20]. At present, the TS test is well accepted as a method for adaption to reduce skeletal muscle use [17]. In this study, we investigated the effect of eldecalcitol in TS-induced disuse muscle atrophy in mice.

## Materials and methods

### Animal experiments

C57BL/6J male mice aged 6 weeks were purchased from Hangzhou Ziyuan Laboratory Animal Technology Co., Ltd. (China). The mice were acclimatized and maintained in a temperature-controlled environment, humidity of 55% to 70%, and a 12-h/12-h dark/light cycle, with access to water and standard rodent chow ad libitum as recommended by the Animal Welfare Act regulations and the Guide for the Care and Use of Laboratory Animals [24]. According to their body weights, the mice were randomized into 4 groups (*n* = 8): control group (vehicle), TS group (vehicle), TS + eldecalcitol low dose (3.5 ng twice per week), and TS + eldecalcitol high dose (5 ng twice per week). The mice were administered with either vehicle (0.8% ethanol, 0.1% Tween 80 in phosphate-buffered saline [PBS]) or eldecalcitol by intraperitoneal injection twice a week, as per the group they were assigned, for 3 weeks [23]. This study was approved by the Animal Ethics and Experimental Safety Committee of Fudan University (SYXK2020-0032).

### Tail suspension in mice

To induce disuse muscle atrophy, the mice were suspended individually in special cages for 21 consecutive days, as elaborated in the previous reports [20, 39]. Briefly, one end of the hinge was connected to the tail by sticking the medical tape, and the other end was connected to the top of the cage. The length of the rope was adjusted to allow free movement of the animal on its forelegs and tilt its body 30 °C to 40 °C horizontally. All mice drank freely and received 8 g of standard rodent food every day. The mice were treated as described earlier for 21 days. The following days after the last treatment, the mice were euthanized and dissected to collect tissues for further analysis.

### Body weight, hind limb muscle weights, and grip strength

Wet muscle weights were recorded immediately after tissue excision. Body weight and muscle weights of the gastrocnemius (GAS), tibialis anterior, and soleus of each mouse were measured by a digital balance. The muscle grip strength of the hind limb was measured by a grip strength meter (KW-ZL Grip Strength Meter, KEW BASIS, Nanjing, China) every week. Forepaws of mice were grasped by a wire grid while being lifted by their tails and their posture was kept parallel to the table surface. A gentle pull at the rate of 2 cm/s was continuously applied to the mice’s tails until the grip was released. The maximum force exerted was measured in grams. The measurements were repeated to collect replicates of data per mouse, and the grip strength was analyzed for each mouse based on the maximum value.

### Histological analysis

The GAS muscles collected from the hind limb of the mice were fixed using 4% paraformaldehyde in PBS, pH 7.4 for 24 h at 4 ^°^C. Following this, the samples were embedded in paraffin and cut into 10 μm sections. These tissue sections were subjected to a series of regular histological processes of deparaffinizing, rehydrating, and counterstaining with hematoxylin/eosin (H&E). The tissue slides from all groups were screened under a microscope (E800; Canon, Tokyo, Japan), and the images were captured using a CCD camera. ImageJ software (NIH ImageJ system, Bethesda, MD, USA) was used to analyze the images and to calculate cross-sectional areas (CSAs) of myofibers.

### Micro-computed tomography

Micro-computed tomography (CT) was performed as described in our previous study [41]. Femurs were fixed in 4% paraformaldehyde for 12 h and transferred to 75% ethanol at 4 °C for analyzing bone microarchitecture by micro-CT at the resolution of 8.96 μm, voltage of 50 kV, and current of 450 mA. The scanned images were analyzed by SCANCO evaluation software to determine the density and distance parameters, including bone volume (BV), tissue volume (TV), bone/tissue volume (BV/TV), bone surface/total tissue volume (BS/TV), bone surface/total bone volume (BS/BV), trabecular number (Tb.N), trabecular thickness (Tb.Th), trabecular separation (Tb.Sp), trabecular pattern factor (Tb.Pf), and structure model index (SMI).

### Oxidative stress makers analysis

We determined the levels of malondialdehyde (MDA), superoxide dismutase (SOD), glutathione peroxidase (GSH-Px), and catalase (CAT) activities as indicators of oxidative stress in GAS muscle and serum after 21 days of treatment with vehicle or eldecalcitol. The mice were killed by cervical dislocation. The GAS muscle of the hind limb was dissected, weighed, and immediately frozen in liquid nitrogen. The GAS homogenate was centrifuged at 12,000 rpm at 4 °C for 15 min, and the supernatant was collected and frozen at − 20°C until the day of assay. For serum preparation, blood samples were collected and centrifuged at 3000 rpm for 15 min. Using the respective detection kits (MDA: A003-1; SOD: A00-1; GSH-Px: A005; CAT: A007, Jiancheng Bioengineering Ltd., Nanjing, China), the assays were performed strictly according to the manufacturer’s instructions.

### Cell culture

The C2C12 cells were purchased from the Cell Bank of the Chinese Academy of Sciences (Shanghai, China). To induce differentiation, C2C12 cells were plated in a 6-well plate with Dulbecco’s modified containing 4.5 g/L glucose (DMEM) (Gibco) growth media supplemented with 10% fetal bovine serum, 1% penicillin, and streptomycin at 37 °C in a humidified atmosphere of 95% air and 5% CO_2_. When C2C12 cells reached 70% to 80% confluency, the medium was changed to DMEM with 2% horse serum (Thermo Fisher Scientific, CA, USA) in the differentiation medium. After 5 days of treatment with DMEM containing 2% horse serum, the C2C12 myoblasts were differentiated into myotubes. Cells were incubated with eldecalcitol at 0.1, 1, and 10 nM, whereas control and tumor necrosis factor (TNF)-α control were incubated with the vehicle. To induce muscle atrophy in vitro, the TNF-α control and eldecalcitol cells were incubated with 100 ng/mL TNF-α in differentiation medium for 24 h. Cells were harvested or used for morphological analysis after incubation.

### C2C12 myotubes diameter measurement

Differentiation in C2C12 myoblasts cultures was induced by 2% horse serum (Hyclone, Chicago, IL, USA) in DMEM. After respective treatment and atrophy induction in vitro, the cells were washed and harvested for Western blotting or fixed in ethanol for immunostaining. The images of cells stained for myotubes were scanned under a microscope using a digital camera mounted on a Nikon Ti microscope. The myotubes diameters were measured using NIS Elements BR 3.00 software (Nikon, Tokyo, Japan). The myotube diameters were quantified from the diameters of 3 randomly selected sites of at least 100 myotubes by ImageJ software.

### Western blot

A radioimmunoprecipitation assay (RIPA) lysis buffer and a Nuclear and Cytoplasmic Protein Extraction Kit (P0027) purchased from Beyotime were used to lyse the total protein and extract nuclear proteins supplemented with protease inhibitors. The protein concentration in the supernatant was measured using a piccinic acid assay kit (Pierce, Rockford, IL, USA). An equal amount of protein (20–30 μg) was used to electrophoresis each sample on the 10–15% sodium dodecyl sulfate-polyacrylamide gel and transferred to the polyvinylidene fluoride membrane. Before incubating with the first antibody, first seal the membrane with BSA (5%) under RT for 1 h. The membrane was washed with Tris-buffered saline containing 0.1% Tween 20 detergent and then reacted with the corresponding secondary antibody for 45 to 60 min. The immunoblots were developed with enhanced chemiluminescence plus reagent, and the results were quantified with laboratory image version 2.7.1.

### Immunofluorescence

After 48 h of treatment with eldecalcitol 10 nM, C2C12 myotubes were exposed to 100 ng/mL of TNF-α for 24 h. A 30-min permeabilization with PBS containing 0.2% Triton X-100 was applied to the spheroids after washing 2 times with PBS. The cells were again rinsed 3 times with PBS for 5 min and blocked for 30 min in PBS with 5% bovine serum albumin. Subsequently, the C2C12 cells were incubated with the primary antibody overnight, and then incubated with the fluorescent secondary antibody for 2 h. In addition, the nuclei were counterstained with a blue DAPI stain.

### Co-immunoprecipitation

After the indicated treatments, the collected cells were lysed on ice for 15 min in radioimmunoprecipitation assay lysis buffer. The supernatant was collected after centrifugation at 14,000 rpm for 10 min and incubated with protein agarose A/G beads (abs955, absin, Shanghai, China) at 4 °C for 60 min to reduce nonspecific binding. Clarified lysate (500-μg protein) was incubated overnight at 4 °C with nonspecific Immunoglobulin G(2 g), mouse anti-VDR antibody (Santa Cruz) overnight on a rotating plate. Afterward, protein A/G agarose beads were added to each sample and incubated at 4 °C for another 2 h. Following this, the precipitate was washed 3 times with a washing buffer, and an equal amount of protein from each sample was subjected to Western blot analysis.

### RNA interference

Individual RNA siRNAs specific for VDR were obtained from RiboBio (Guangzhou, China) as negative controls. C2C12 cells were maintained in a differentiation medium for 4 days in 6-well plates. The myotubes were transfected with Lipofectamine RNAiMAX transfection reagent (Invitrogen, USA) according to the manufacturer’s instructions. The base sequence for si-VDR: GGCAGCCAAGACTACAAT.

### Statistical analysis

Statistical analysis was performed using SPSS 21.0 software (Chicago, IL, United States). The data were expressed as the mean ± standard error of the mean. In vitro cell experiments were performed in replicates of 4 to reduce error in sampling. One-way ANOVA was used to analyze the significance of differences between experimental groups. *P* < 0.05 was considered as statistically significant.

## Results

### Eldecalcitol restored the TS-induced muscle loss and enhanced skeletal muscle function

**Fig. 1:**
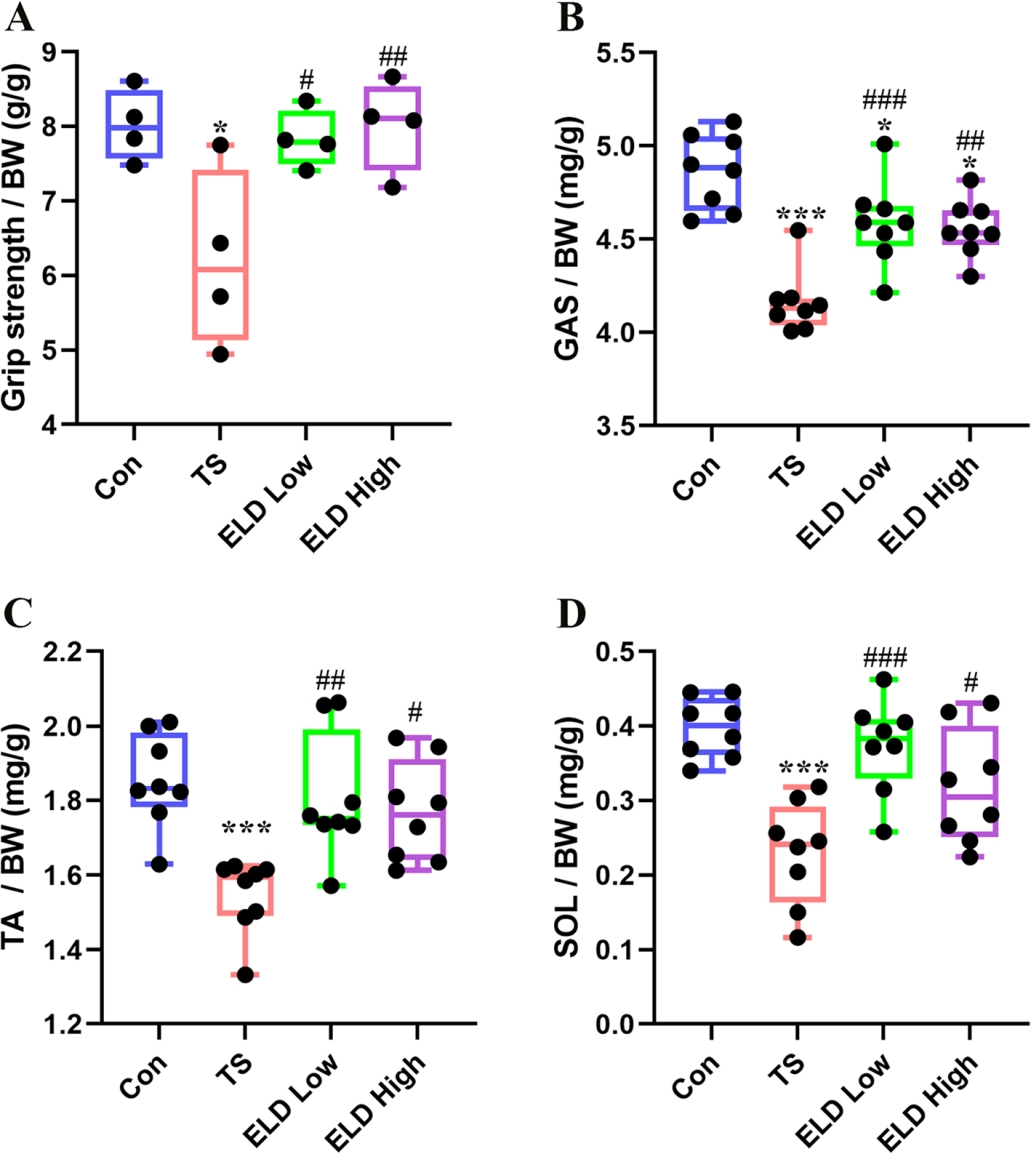
Eldecalcitol improved the lower limb weight reduction and muscle function reduction caused by TS. **a** Grip strength. **b** GAS muscle. **c** TA muscle. **d** SOL muscle. Values are presented as mean ± SEM (*n* = 8). *P < 0.05; **P < 0.01; ***P < 0.001 vs. Con; ^#^*P* < 0.05; ^##^*P* < 0.01; ^###^*P* < 0.001 vs. TS. BW, body weight; Con, control; GAS, gastrocnemius; TA, tibialis anterior; TS, tail suspension; ELD, eldecalcitol; SEM, standard error of mean; SOL, soleus

**Fig. 2:**
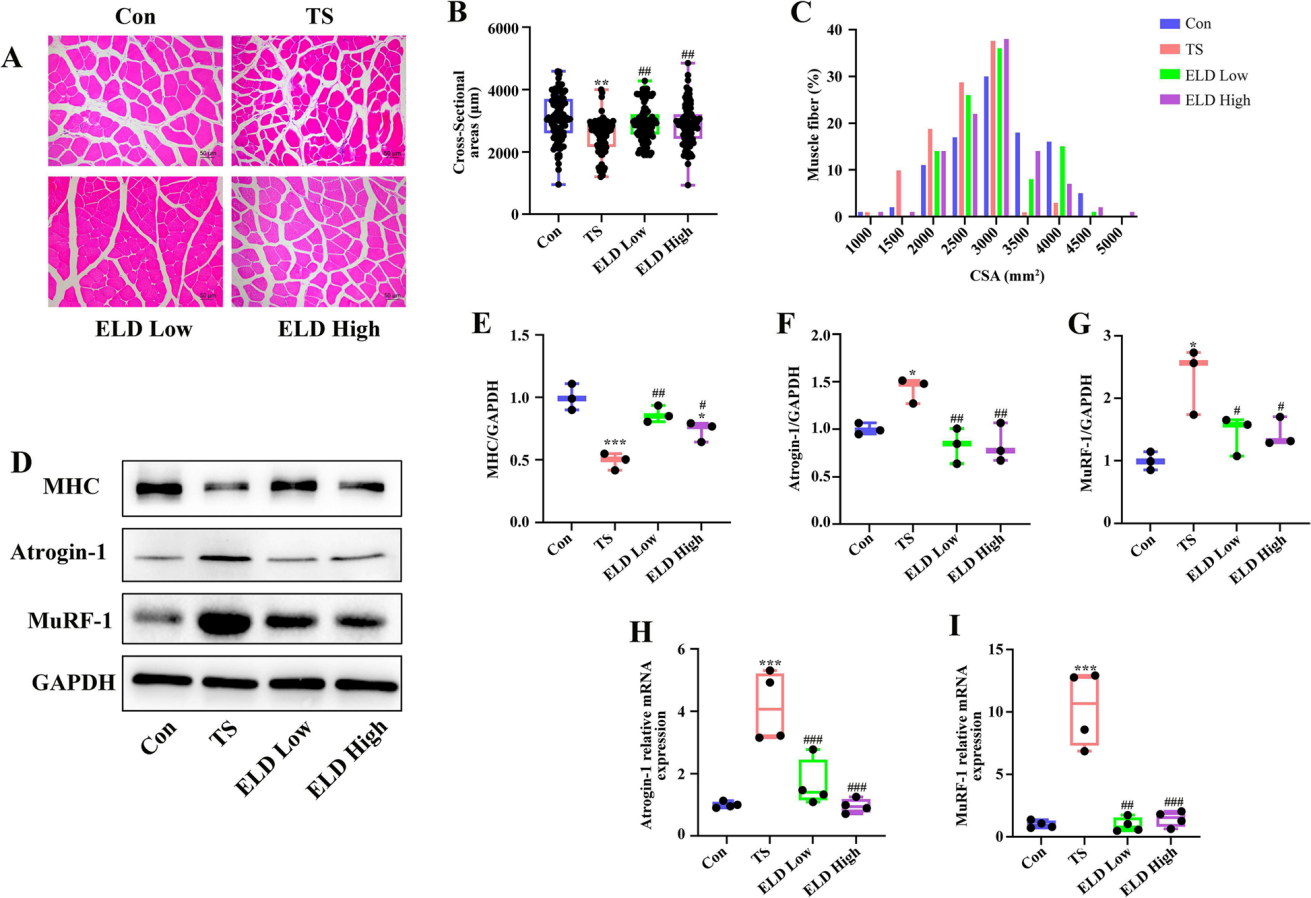
Eldecalcitol inhibited the reduction of muscle cross-sectional area and the expression of muscle atrophy protein markers. **a**–**c** H&E stating of GAS muscle and analysis of muscle cross-sectional area. **d**–**g** Eldecalcitol decreased the protein expression levels of Atrogin-1 and MuRF-1 and increased the protein expression levels of MyHC. **h**–**i** mRNA levels of Atrogin-1 and MuRF-1 by qPCR. Values are presented as mean ± SEM (*n* = 8). *P < 0.05; **P < 0.01; ***P < 0.001 vs. Con; ^#^*P* < 0.05; ^##^*P* < 0.01; ^###^*P* < 0.001 vs. TS. Con, control; CSA, cross-sectional area; GAPDH, glyceraldehyde 3-phosphate dehydrogenase; ELD, eldecalcitol; TS, tail suspension; MyHC; myosin heavy chain; SEM, standard error of mean

We investigated the effects of eldecalcitol on skeletal muscle and osteoporosis in disuse muscle atrophy in a mouse TS model. The relative hind limb grip strength was significantly decreased by 22.49% during TS while administering eldecalcitol-mitigated TS-induced muscle wasting (Fig. 1a). Skeletal muscle mass showed improvement with eldecalcitol supplement. We used a muscle weight to body weight (mg/g) standardized ratio to identify muscle wasting. In the TS group, the muscle weights of the GAS (Fig. 1b), TA (Fig. 1c), and SOL (Fig. 1d) were significantly decreased by 14.39%, 16.73%, and 42.34%, respectively. Nevertheless, eldecalcitol treatment helped to recover this loss and brought the muscle weight back to the levels of the control group. The CSA of GAS muscle was reduced to about 18.59% after TS compared with the control group. Nonetheless, the decrease was significantly reversed up to the levels of the control group (94.1%) by the administration of eldecalcitol (Fig. 2a–c). Taken together, these effects of eldecalcitol result in increased muscle strength and function. Furthermore, the expression of muscle atrophy markers, Atrogin-1 and MuRF-1, in the TS group was markedly upregulated by 42.1% and 134.6%, respectively, but eldecalcitol treatment suppressed the levels by 41.52% and 38.79%, respectively (Fig. 2d, f, and g). Consistent with the protein expression level, the mRNA expression level of Atrogin-1 and MuRF-1 was significantly down-regulated after ELD application (Fig. 2h, i). Eldecalcitol also restored the expression levels of myofibrillar proteins, such as MHC, which were decreased in the GAS muscle of mice after TS (Fig. 2d, e).

### Effects of eldecalcitol on the bone loss of TS mice

**Fig. 3:**
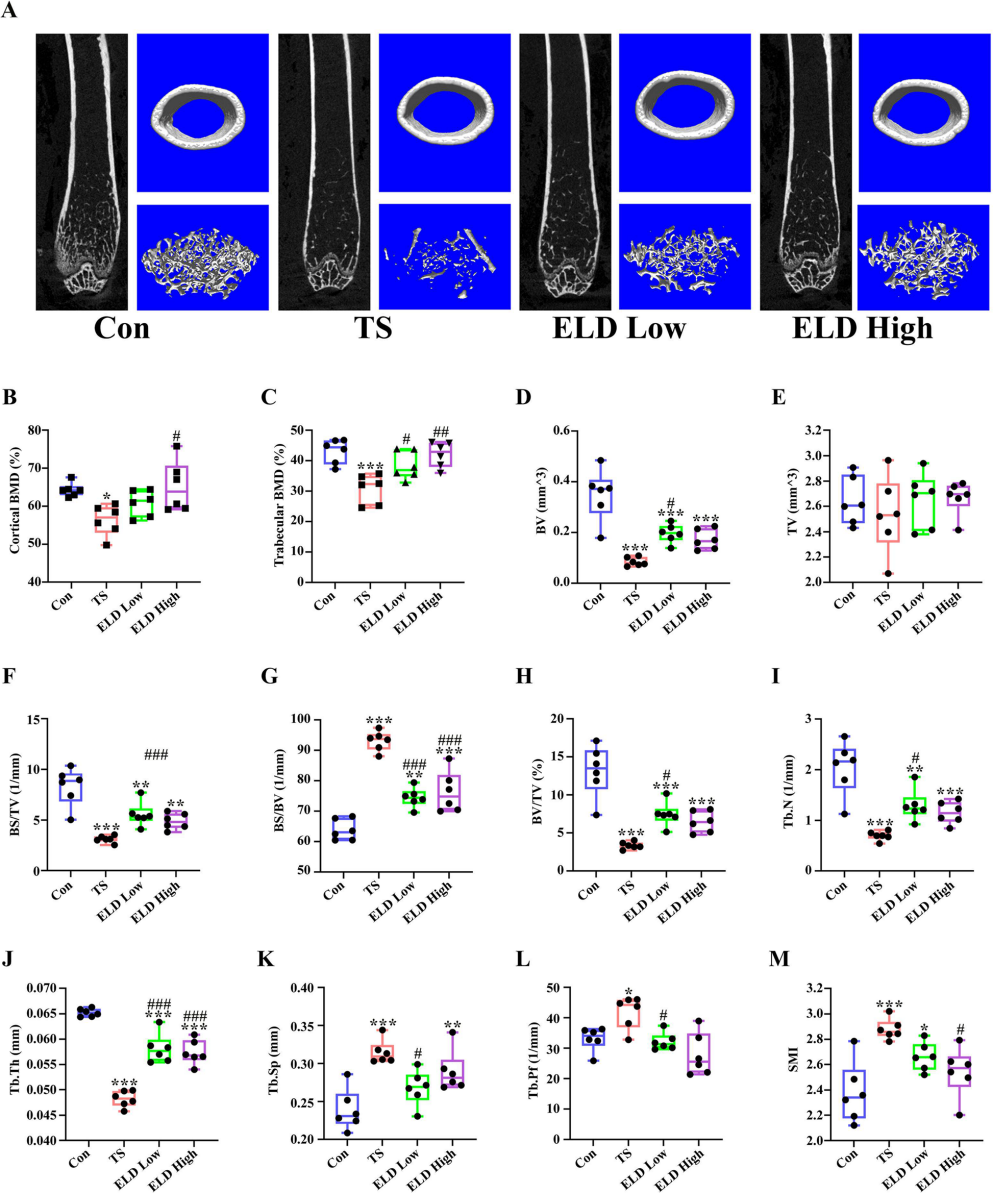
Effect of eldecalcitol administration on TS-induced bone loss. **a** Bone phenotypes. **b** Cortical BMD. **c** Trabecular BMD. **d** BV. **e** TV. **f** BS/TV. **g** BS/BV. **h** BV/TV. **i** Tb.N. **j** Tb.Th. **k** Tp.Sp. (l) Tb.Pf. **m** SMI. Values are presented as mean ± SEM (*n* = 8). *P < 0.05; **P < 0.01; ***P < 0.001 vs. Con; ^#^*P*< 0.05; ^##^*P*<0.01; ^###^*P* < 0.001 vs. TS. BMD, bone mineral density; BS/BV, bone surface/total bone volume; BS/TV, bone surface/total tissue volume; BV, bone volume; BV/TV, bone/tissue volume; Con, control; ELD, eldecalcitol; TS, tail suspension; SEM, standard error of mean; SMI, structure model index; Tb.N; trabecular number; Tb.Pf, trabecular pattern factor; Tb.Th, Trabecular thickness; Tp.Sp, trabecular separation; TV, tissue volume

We characterized trabecular and cortical bone across all groups using Micro-CT. The bone phenotypes of all groups are presented in Fig. 3a. Cortical BMD and trabecular BMD of the distal femur were significantly decreased by 12.37% and 28.51%, respectively, after 21 days of TS as compared with control. Loss of cortical and trabecular BMD was reversed by the administration of eldecalcitol (Fig. 3b, c). The administration of eldecalcitol partly restored the BV in TS mice (Fig. 3d) but TV did not differ between the 4 groups (Fig. 3e). Thus, the decrease in BV/TV caused by TS was significantly improved by 122.66% in low-dose and 92.87% in high dose after the supplementation of eldecalcitol compared with the TS group (*P* < 0.05) (Fig. 3h). BS/TV, Tb.N, and Tb.Th were markedly decreased by 62.56%, 65.92%, and 26.11%, respectively, in the TS group after 21 days of TS compared with the control group (*P* < 0.001) (Fig. 3f, i, and j). However, their levels were partly restored by 78.22%, 85.68%, and 19.04%, respectively, in the eldecalcitol low-dose group. In the TS group, there was an increase in the BS/BV (45.81%), Tb.Sp (31.82%), Tb.Pf (26.61%), and SMI (21.06%) compared with the control group (Fig. 3g, k–m). However, treatment with eldecalcitol partially reduced these effects compared with the TS group. Collectively, these results imply that the eldecalcitol supplement could partly reduce TS-induced bone loss.

### Eldecalcitol suppressed oxidative stress in TS mice

**Fig. 4:**
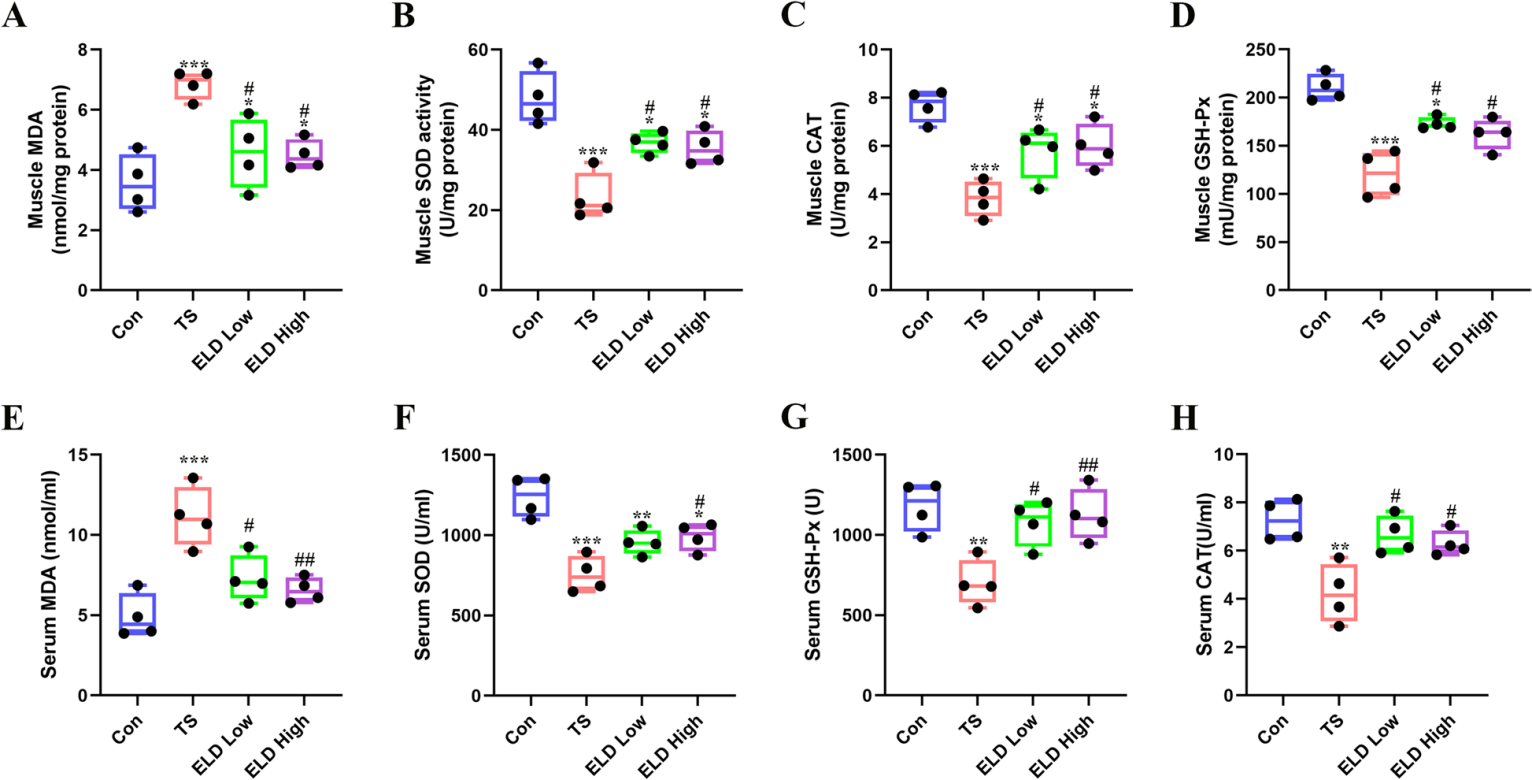
Eldecalcitol suppressed oxidative stress in TS mice. **a**–**d** MDA, SOD, GSH-Px, and CAT in GAS muscle and in serum (**e**–**h**). Values are presented as mean ± SEM (*n* ≥ 3). *P < 0.05; **P < 0.01; ^***^P < 0.001 vs. Con; ^#^*P* < 0.05; ^##^*P* < 0.01; ^###^*P* < 0.001 vs. TS. CAT, catalase; Con, control; ELD, eldecalcitol; GSH-Px, glutathione peroxidase; MDA, malondialdehyde; TS, tail suspension; SEM, standard error of mean; SOD, superoxidase dismutase

Previous studies have reported that disuse muscle atrophy could cause oxidative stress [31]. Therefore, we examined the influence of eldecalcitol on TS-induced oxidative stress. The antioxidant defense system of GAS muscle and serum was altered in the TS group as indicated by the lower levels of SOD, GSH-Px, and CAT in GAS muscle (decreased by 51.38%, 42.48%, and 50.31%) and in serum (decreased by 39.04%, 40.53%, and 41.93%) respectively (Fig. 4b–d, f–h). However, the impaired antioxidant defense system was reversed by eldecalcitol treatment. Furthermore, in the TS group, the levels of MDA were dramatically increased in GAS muscle by 92.42% and serum by 126.58% after 21 days of muscle disuse, which were diminished while treating with eldecalcitol (Fig. 4a, e).

### Eldecalcitol inhibits myotube atrophy in a concentration-dependent manner in vitro

**Fig. 5:**
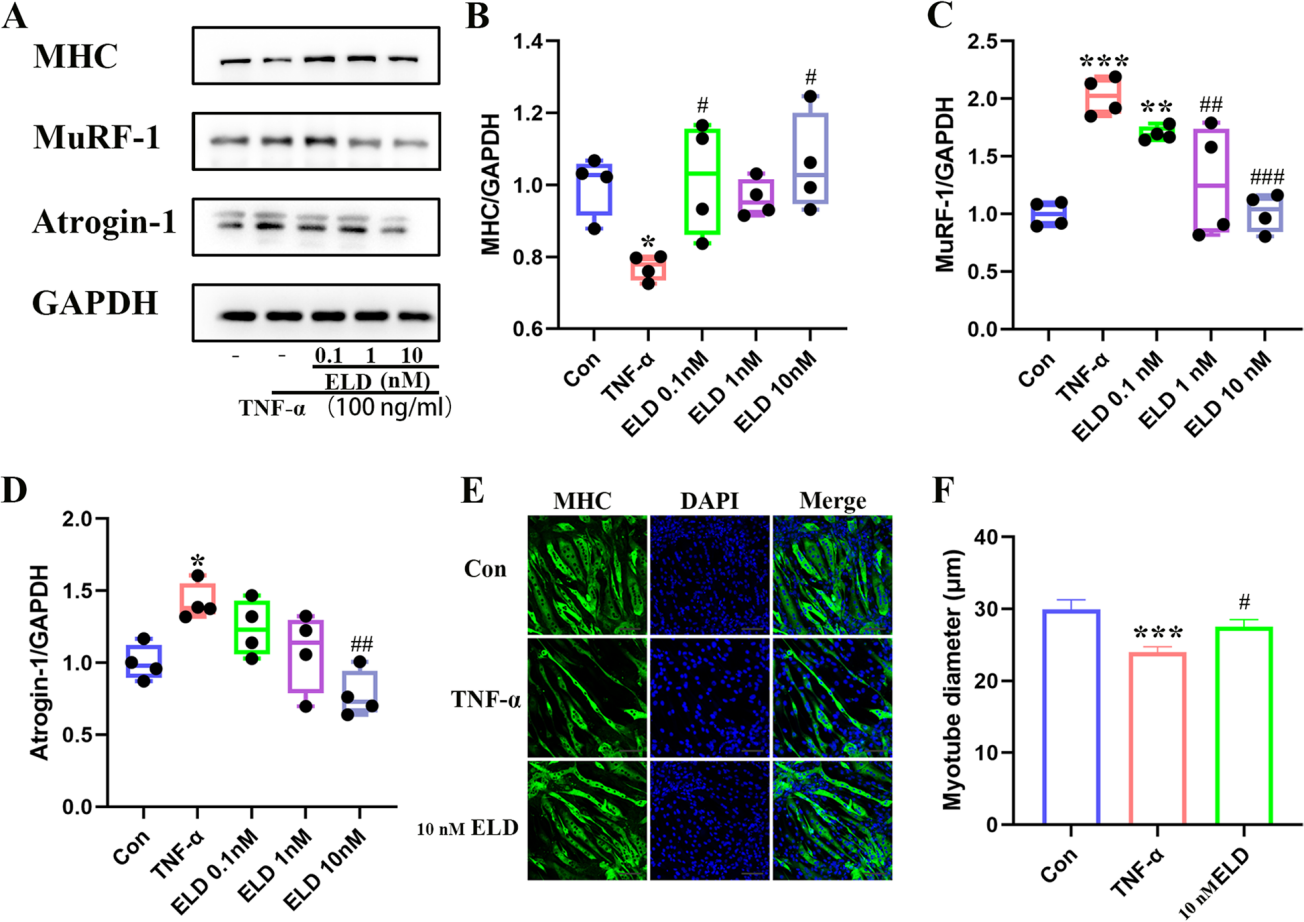
Effects of eldecalcitol on myotube atrophy induced by TNF-α in a concentration-dependent manner. **a**–**d** Western blotting indicated that 10 nM eldecalcitol repressed muscle atrophy caused by 100 ng/ml TNF-α in C2C12 myotubes. **e**–**f** Immunofluorescence showed that 10 nM eldecalcitol increased myotube diameter. Values are presented as mean ± SEM, *n* ≥ 3. *P < 0.05; **P < 0.01; ***P < 0.001 vs. Con group; ^#^*P* < 0.05; ^##^*P* < 0.01; ^###^*P* < 0.001 vs TNF-α group. Con, control; ELD, eldecalcitol; GAPDH, glyceraldehyde 3-phosphate dehydrogenase; SEM, standard error of mean

In differentiated C2C12 myotubes, the addition of TNF-α elevated the levels of Atrogin-1 and MuRF-1 and decreased the levels of MHC (Fig. 5a–d) compared with the control cells. Eldecalcitol inhibited TNF-α–induced myotube atrophy in a concentration-dependent manner. Eldecalcitol at 10 nM significantly inhibited MuRF-1 (*P* < 0.001) and Atrogin-1 (*P* < 0.01) and restored the markers of atrophy to the level close to the control group. In addition, we confirmed that TNF-α induced a reduction in the diameter of myotube that was counteracted by eldecalcitol by improving the myotube diameter, as visualized by immunofluorescence (Fig. 5e, f).

### Eldecalcitol reduces the expression of P65 and P52 and nuclear location of P65 in response to TNF-α

**Fig. 6:**
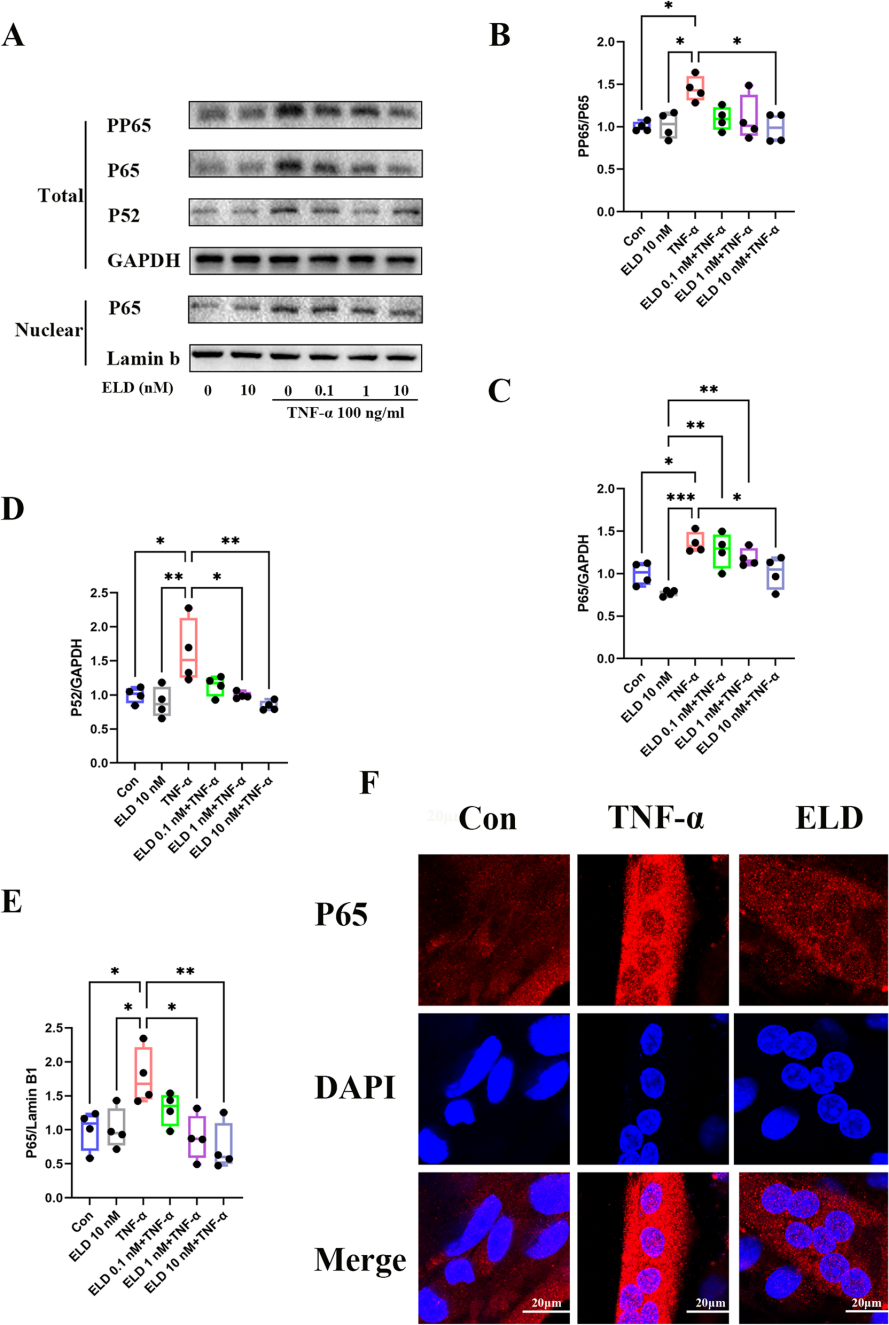
Eldecalcitol reduces the expression of P65 and P52 and nuclear location of P65 in response to TNF-α. **a**–**e** Total protein levels of PP65, P65, and P52 were measured by Western blot analysis and nuclear protein level of P65. **f** Representative pictures of immunofluorescence showed that eldecalcitol reduced P65 nuclear translocation. Values are presented as mean ± SEM, *n* ≥ 3. *P < 0.05; **P < 0.01; ***P < 0.001 vs. control group; ^#^*P* < 0.05; ^##^*P* < 0.01; ^###^*P* < 0.001 vs TNF-α group. Con, control; ELD, eldecalcitol; GAPDH, glyceraldehyde 3-phosphate dehydrogenase; SEM, standard error of mean

The NF-κB signaling pathway plays a crucial role in muscle atrophy. In our study, the expression levels of total P65 and P52 components of NF-κB were elevated with 100 ng/mL TNF-α treatment in C2C12 cells (Fig. 6a, c, and d). Likewise, the expression of PP65/P65 was also upregulated, implying that TNF-α activated NF-κB signaling pathway (Fig. 6b) in myotubes. Nevertheless, the nuclear total P65 expression was reduced in myotubes with prior eldecalcitol treatment (Fig. 6a, e). Our data showed eldecalcitol inhibited the expression of P65 and P52 and the activation of P65 phosphorylation. P65 immunostaining and microscopy revealed that eldecalcitol decreased the nuclear translocation of P65 induced by TNF-α (Fig. 6f).

### VDR helps mediate the anti-atrophy effects of eldecalcitol in TNF-α treated myotubes

**Fig. 7:**
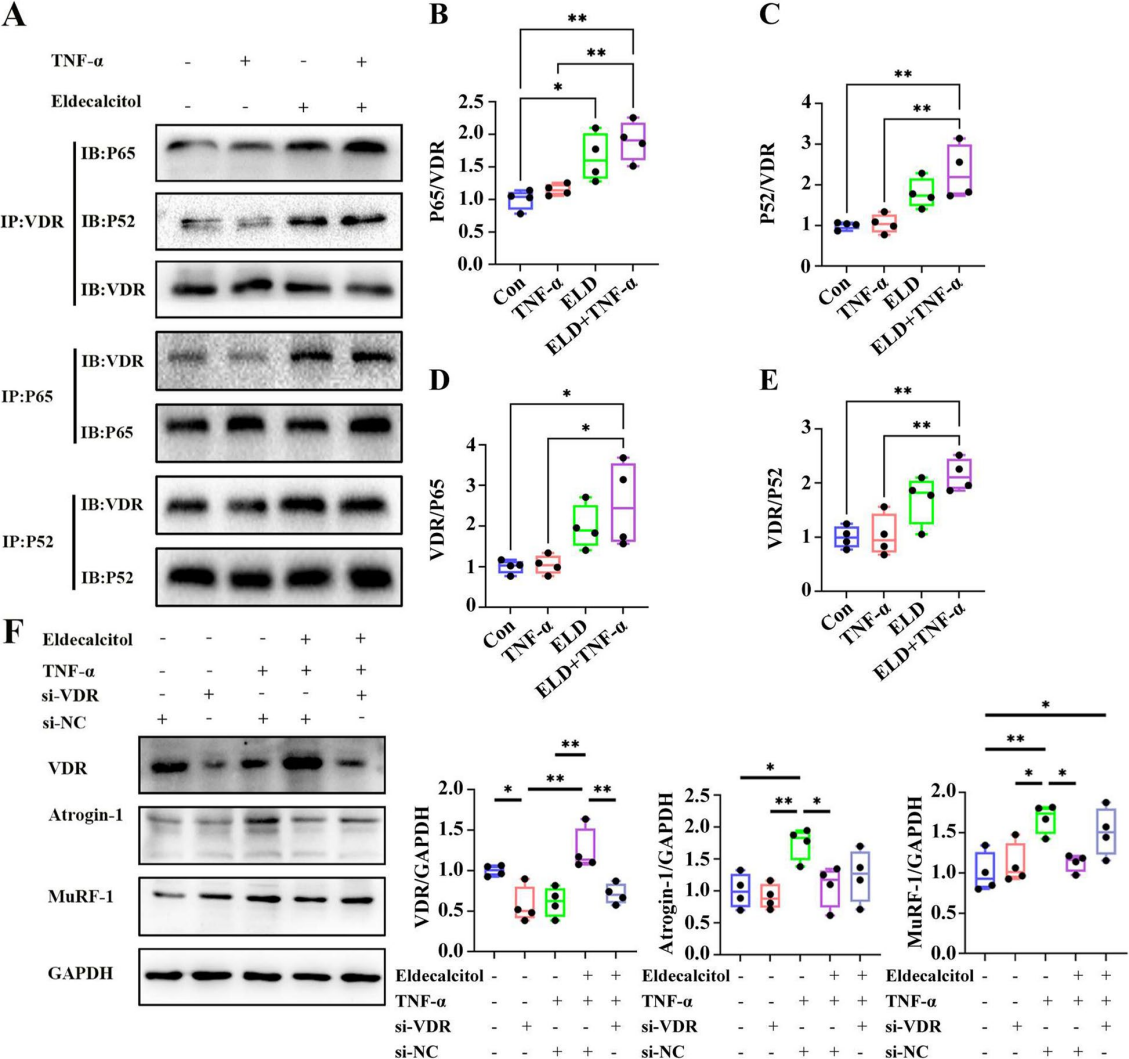
VDR helps mediate the anti-atrophy effects of eldecalcitol in TNF-α treated myotubes. **a**–**c** Eldecalcitol increased the interaction of VDR with P65 and P52. C2C12 myotubes were stimulated with TNF-α (100 ng/ml) for 24 h in the presence or absence of eldecalcitol (10 nM). **d**–**g** Eldecalcitol-mediated anti-atrophy effects were partly blunted by VDR gene silencing in myotubes exposed to TNF-α. Values are presented as mean ± SEM, *n* ≥ 3. *P < 0.05; **P < 0.01; ***P < 0.001. Con, control; ELD, eldecalcitol; GAPDH, glyceraldehyde 3-phosphate dehydrogenase; SEM, standard error of mean, VDR, vitamin D receptor

So far, our data have suggested that eldecalcitol prevents TNF-α induced NF-κB nuclear location and inhibits the expression of muscle atrophy markers through VDR. To further confirm this notion, we first performed co-immunoprecipitation with an antibody against VDR. We observed a much higher interaction between P65 and VDR as well as between P52 and VDR in myotubes stimulated with TNF-α and treated with eldecalcitol (Fig. 7a–e). This suggested that eldecalcitol promotes VDR binding to P65 and P52 in TNF-α treated myotubes. The role of VDR in eldecalcitol on muscle atrophy was further verified by VDR knockdown experiments. The data presented in Fig. 7f indicated that the TNF-α induced upregulation of atrophy markers, Atrogin-1, and MuRF-1 was dramatically reduced by eldecalcitol. The inhibitory effects of eldecalcitol, however, were partly abolished by silencing the VDR gene. These data demonstrate that VDR signaling is required for eldecalcitol-mediated anti-atrophy effects.

## Discussion

Disuse muscle atrophy is a common clinical problem. Although this silent skeletal disease occurs at all ages and genders, its prevalence is higher in older adults [4, 25]. Atrophy can occur in various diseases and conditions and mainly results from excessive protein breakdown in addition to declining protein synthesis leading to muscle wasting and weakness [5]. Osteoporosis is strongly associated with bone mass reduction and skeletal muscle loss, especially in older adults [4]. Involutional loss of functional muscle motor units combined with the high prevalence of osteoporosis in the geriatric population increases the risk of debilitating postural changes [34]. Examining the physiology and mechanisms underpinning muscle disuse atrophy involves invasive muscle biopsy sampling, because of which rodent models mimicking the different forms of human muscle disuse atrophy are used.

In this study, we observed that treatment with eldecalcitol reversed the TS-induced muscle mass loss and muscle function as evidenced by the improved grip strength (at low dose, *P* < 0.05 and high dose *P* < 0.01) and improved standardized muscle strength to body weight ratio (*P* < 0.05 to *P* < 0.001) compared to the TS group. In a previously published study [36], a dose-dependent investigation involving eldecalcitol at dosages of 0.05, 0.1, and 0.2 μg/kg exhibited significant enhancements in BMD and mechanical properties within an ovariectomized rat model. In Japan, eldecalcitol has undergone clinical trials for the treatment of osteoporosis at daily doses of 0.5, 0.75, or 1.0 μg/day [9, 21]. Based on this evidence, we administered eldecalcitol doses to mice, ranging from 30 to 50 ng/kg, following a 3-day-per-week regimen. Quantification of muscle CSA is another commonly used parameter in the assessment of muscle atrophy and in our study, TS-induced reduction in CSA of GAS muscle was significantly repealed by eldecalcitol supplementation (*P* < 0.01) compared to the TS group. Studies using animal models in the setting of hind limb unloading have revealed that muscle loss can also induce bone loss. The primary adaptations of bone to disuse atrophy are demineralization and loss of trabecular and cortical bone [1]. However, eldecalcitol supplementation strongly inhibited bone loss and increased bone mineral density in our study. Furthermore, eldecalcitol also reduced ubiquitin-mediated protein degradation in skeletal muscle as was apparent in the expression levels of Atrogin-1 and MuRF-1. The ubiquitin-proteasome is the primary regulator of muscle protein degradation in which Atrogin-1 and MuRF-1 direct the polyubiquitination of proteins causing proteolysis. Atrogin-1 and MuRF-1 are increased by immobilization or denervation by myostatin/TGF-β signaling [8]. It has been shown that Atrogin1/MAFbx is upregulated in response to TNF-α and is associated with the activation of p38 that stimulates the expression of the ubiquitin ligase atrogin1/MAFbx in skeletal muscle [18]. We observed that eldecalcitol inhibited the effects of TNF-α on the levels of Atrogin-1 and MuRF-1 in a dose-dependent manner and restored the diameters of myotubes close to that of the control group.

There is a clear link between oxidative stress and disuse muscle atrophy, wherein reactive oxygen species (ROS) play a critical role in the cell signaling pathways that regulate protein synthesis and degradation [16, 30]. Prolonged periods of muscle disuse have been reported to induce higher levels of ROS in skeletal muscle fibers, resulting in oxidative injury and fiber damage that is characteristic of muscle disuse atrophy [22]. We studied the effects of eldecalcitol on lipid peroxidation and oxidative damage in TS mice. Eldecalcitol treatment alleviated TNF-α induced oxidative stress by decreasing ROS generation by MDA and by increasing the activities of SOD, GSH-Px, and CAT which were all statistically significant. Accumulating evidence suggests that vitamin D analog supplementation has a protective effect on skeletal muscle against immobilization-induced atrophy [28].

The actions of eldecalcitol, a vitamin D analog, are mediated by VDR, a ligand-activated transcription factor that controls gene expression [27]. The exact mechanism of how vitamin D exerts its anti-atrophy effects via VDR remains unclear. NF-κB is a prominent signaling pathway linked to skeletal muscle loss in various pathophysiological conditions. Activation of NF-κB in skeletal muscle has been reported to cause the degradation of specific muscle proteins, induce inflammation, and inhibit the regeneration of myofibers during atrophy [14]. In our study, co-immunoprecipitation experiments showed that eldecalcitol promotes VDR binding to NF-κB components; P65 and P52 inhibiting their expression besides inhibiting P65 phosphorylation and its relocation to the nucleus thereby attenuating the downstream signaling. In the VDR knockdown experiments, these effects of eldecalcitol were blunted, suggesting that eldecalcitol exerts its atrophy preventive effects through NF-κB signaling mediated via VDR.

This study has a few limitations. Although we showed that VDR is crucial for the therapeutic effect of eldecalcitol, we have yet to study in detail the influence of VDR polymorphism on eldecalcitol. This may throw light on the purported influence of VDR genetic variants on the therapeutic efficacy of eldecalcitol as studies have pointed out an association between VDR genetics variants and osteoporosis [12, 43]. Therefore, further studies are required to confirm these findings and expand the knowledge in the direction of VDR polymorphism and the effects of eldecalcitol in preventing disuse muscle atrophy.

## Conclusions

In summary, our study showed that TS-induced atrophy resulted in muscle and bone loss. Eldecalcitol significantly improved muscle loss and function and bone volume in disuse muscle atrophy. The results of this study indicate that eldecalcitol exerted these effects via alleviating oxidative stress and the NF-κB signaling pathway. Hence, eldecalcitol supplementation could be a potentially effective therapeutic strategy for preventing and reversing disuse muscle atrophy, warranting its evaluation in the clinic.

## Data Availability

The data used to support the findings of this study are presented here. Any further requirements of the data are available from the corresponding author upon request.
